# Supplementary material for: Tumorigenicity decrease in Bcl-xL deficient MDCK cells ensuring the safety for influenza vaccine production
Source: PLoS One. 2024 Dec 16;19(12):e0311069. doi: 10.1371/journal.pone.0311069 (PMC11649150; doi:10.1371/journal.pone.0311069)
Supplement: S1 File — (ZIP) [file pone.0311069.s002.zip › S2_File/3_KEGG_enrichment/pathwaymap/B_vs_A.maps/map04340.html]

KEGG Pathway: Hedgehog signaling pathway
